# Book Review

**Published:** 1935

**Authors:** 


### Reviews of Books

**Collected Papers of St. Mark's Hospital, London, including a History of the Hospital.** Edited by The Medical Committee. Pp. xvi., 440. Illustrated. London: H. K. Lewis & Co. Ltd. 1935. Price 30s.—This beautifully-produced book makes a much wider appeal than the title at first suggests. The history of such a famous hospital is always worth reading. Each paper is written by an author especially experienced and interested in his subject, and the whole collection covers a wide field so comprehensively that the book is a valuable source of reference. Although the views expressed by some of the older masters have in recent years been modified, every paper may be read with profit, and it is interesting to see the way in which progress has been made towards present-day opinion and practice.

**An Anthology of Wit.** By A. E. Roche, M.A. Pp. x., 36. London: H. K. Lewis & Co. Ltd. 1935. Price 1s.—There is little that can properly be called witty in the quotations chosen for this little collection, if we except a few well-known and cynical epigrams. But the Introduction, devoted by the author to a defence of the unexpurgated edition, is a good example of the unconsciously funny.

**Medical and Other Verses.** By Alex. E. Roche. Pp. x., 92. London: H. K. Lewis & Co. Ltd. 1935. Price 3s. 6d.—Though himself an admirer of Catullus, the author of this little volume has adopted the advice of Horace to let his verses mature for two lustres before giving them to the expectant world. Now that our patience is at length rewarded we find a very pleasant miscellany to entertain us. If Pegasus never soars with this rider, at least he seldom stumbles. The collection falls really into three parts: verses on various subjects suggested by the life of a medical student, sonnets and occasional verses of a sentimental turn, and translations from Catullus and other classical authors. The first are of their very nature light, and though amusing enough, tend to be marred by false rhymes which are not permissible in versification of this nature—hypertrophied, certified ; forward, onward ; torpid, morbid ; injected, infested ; vital, cycle. And surely in sea-sickness the brow is *cold* and sweating, not hot ! The sonnets are fresh and pleasant in thought and musical in expression. It is curious that a classical scholar twice refers to the sirens as alluring by their form instead of by their voices. In the translation the author is particularly happy, especially in his version of Catullus, Ode III., the favourite "Epitaph on Lesbia's Sparrow." Altogether, though not deathless verse, a very pleasant little collection to amuse an idle hour.

**Catechism Series : Anatomy (Head and Neck).** Fourth Edition. By C. R. WHITTAKER, F.R.C.S., F.R.S.E. Pp. iv., 80. Edinburgh : E. & S. Livingstone. 1935. Price 1s. 6d.—This well-known series has reached its fourth edition, a tribute to Mr. Whittaker's celebrated coaching classes in Edinburgh. It is a cram book for examinations on the conventional lines of memorised description ("trace, name, mention, how, what, give"), but we are glad not to find that word beloved by clinicians—"discuss,"—which surely implies an interlocutor. If anatomical examiners still demand detailed and tabulated descriptions of, so to speak, the streets, lanes, pipes and wires of the human city without any consideration of their uses (that is, of functional anatomy) such booklets as these have value, but not an educational one. The student may take it with him on an interrogative walk with his fellow and pass it between them, but I fancy they would seek refreshment after citation of the tabulated list of forty-seven structures met with in a dissection of the anterior triangle of the neck (p. 21). They would have a more useful mental exercise in formulating the neuro-anatomical mechanisms by which they merely walked and talked. An examiner may find a use for it if at a loss in setting the usual paper, as also the surgeon for a quick reminder of points and relations. For trifling criticisms, we would say that even a Duke Elder doesn't care about the exact position to a millimetre or so of the eyeball in its socket (p. 55) ; that a dissector will not find the fascia bulbi to be a continuation of the dural-sheath of the optic nerve (p. 51) ; and that even the proof-reader might have wondered how both the medial and lateral recti could adduct the eyeball (p. 52).

**Catechism Series: Physiology,** Parts 3, 4, 5, Fourth Edition, pp. xii., 228; **Histology,** Third Edition, pp. iv., 76. Illustrated. Edinburgh: E. & S. Livingstone. 1935. Price 1s. 6d. each part.—These booklets contain a fair amount of physiological knowledge imparted in the form of question and answer. It would appear from the serial numbering that they attempt to cover the subject of Physiology in 1,585 brief paragraphs. The standard is somewhat uneven, and, as would be expected in short answers, some of the material is sketchy. Physiology cannot be satisfactorily treated in this form. The only justification for their publication is presumably as a preparation for examinations, but one strongly hopes that students will not be tempted to try to revise their Physiology and Histology in this way. Besides a few errors in the text the information given is sometimes curious. "In general, adrenalin dilates" is a misleading beginning to a summary of the actions of this hormone, and is an example of a dangerous mnemonic. The answer to a question on a convenient form in which to carry a supply of vitamin C is, "Carry dried peas and let them germinate." In the section on the liver the necessity of this organ for life is not mentioned. The section headed Reproduction deals only with embryology; the functions of the sex glands are to be found in a different booklet.

**An Introduction to General Therapeutics.** By H. K. FRY, B.Sc., M.D., D.P.H. Pp. 223. London: Cassell & Co. 1935. Price 6s.—This book is a very successful attempt to provide a concise summary of modern methods of treatment. It is thoroughly up to date in its outlook and deals with the rationale of treatment by rest, exercise, drugs, physiotherapy, immunology, organotherapy, etc. All methods of treatment are described and their value critically considered. It is refreshing to find a book on therapeutics which is not overburdened with drugs and methods which have survived more by nature of their antiquity than from any proved therapeutic effect. In addition, full recognition is paid to the treatment of disease by other means than "a bottle of medicine." It should be of great help, not only to students, but also to newly-qualified practitioners in assisting them to formulate definite and sound ideas on the principles of treatment.

**The Rheumatic Diseases.** By G. D. KERSLEY, M.D. Pp. xvi., 88. Illustrated. London: William Heinemann Ltd. 1934. Price 6s.—This is a concise manual on the subject of Rheumatic Diseases for the Student and Practitioner. Though it contains nothing that is new, it has the merit of being accurate and of not suffering from that unwarranted enthusiasm with regard to treatment which characterizes one or two other works of a similar type. The Student will find here the various types of Arthritis concisely described, and the different methods of treatment clearly outlined.

**Mentality and the Criminal Law.** By O. C. M. DAVIS, M.D., D.Sc., and F. A. WILSHIRE. Pp. vi., 168. Bristol: John Wright & Sons Ltd. 1935. Price 5s.—This small volume starts with at least three advantages: it is very readable, it is concise, and as far as one's memory goes no other book covers quite the same ground. To general practitioners the appendix, giving a summary of statutory enactments relating to various types of mental abnormalities, will be found especially useful. The question of criminal responsibility before and since MacNaghten's rule nearly a hundred years ago has always been a much debated one. Two short passages in the report of Lord Atkin's Committee very admirably sum up the present position. They are as follows: "A crime no doubt implies an act of conscious volition; but if a person intends to do a criminal act, has the capacity to know what the act is, and to know he ought not to do it, he commits a crime." "If the mental conditions we have presupposed exist, we think that punishment may be fairly inflicted. It is probable that the offender and others will be deterred. On the other hand, if the offender tends to escape punishment by means of nicely-balanced doubt upon a diagnosis of uncertain mental conditions, the observance of the law is gravely hindered." These observations might well be incorporated in a future edition of this volume.

**The Hair and Scalp.** By AGNES SAVILL, M.D., M.R.C.P. Pp. vii., 288. Illustrated. London: Edward Arnold & Co. 1935. Price 12s. 6d.—Dr. Agnes Savill is to be congratulated on this book, in which she deals very thoroughly not only with the diagnosis and treatment of diseases of the hair and scalp, but also with the care of the hair. Details are given of the various methods of curling and waving, including the permanent wave. An interesting feature is a chapter by Mr. W. T. Astbury on the molecular structure and elastic properties of hair. One doubts if the medical student will find time to read a book such as this, but it should prove of value to the general practitioner, the dermatologist and the hairdresser. The book is illustrated with photographs of some of the diseases described, and with microphotographs which are well produced. There are many text-books of dermatology, but comparatively few monographs on special diseases of the skin, and one feels that there is scope for addition to the latter group rather than to the former. This is an excellent contribution to dermatological literature, and can be recommended with confidence.

**Physical Signs of Clinical Surgery.** Fifth Edition. By HAMILTON BAILEY, F.R.C.S. Pp. xii. 287. Illustrated. Bristol: John Wright & Sons Ltd. 1935. Price 21s.—This is an extraordinarily good book. That it has been widely appreciated is shown not only by reviews, but also by the fact that five editions have been called for in eight years. It has been brought thoroughly up to date without any great increase in bulk. There are no less than 341 illustrations in a volume of 272 pages, and they are all good and useful. To read this book, or even to look at the pictures, is a liberal education for the practitioner or student in a most important and practical subject.

**An Introduction to Surgery.** Third Edition. By RUTHERFORD MORISON, LL.D., D.C.L., M.D., F.R.C.S., and CHARLES F. M. SAINT, M.D., M.S., F.R.C.S., F.R.A.C.S. Pp. xii. 367. Illustrated. Bristol: John Wright & Sons Ltd. 1935. Price 15s.—The two most attractive features about this little book are the number and excellence of the illustrations and the evidence it gives of the continued activity of a great master and teacher in the surgical world. The idea at which the book aims is to teach principles so that the student can then understand details of technique. This is a most difficult task, and has been more successfully accomplished in some chapters than others. Eighty-six pages are devoted to syphilis, tubercle and malignant disease, whilst fractures, osteitis, appendicitis, arthritis, stricture of the urethra, intestinal obstruction, salivary calculus, endocrine glands, diseases of the breast and carcinoma of the rectum are all grouped together in sixty-eight pages. With many of the opinions expressed we are not in agreement, but all of them require our serious consideration as coming from one of Professor Morison's experience. Some of these are: That amputation should be regarded as the best treatment for tuberculous knee in patients over fifty; that tuberculous abscess should be treated by radical operation involving extensive incisions ; that X-rays can be relied on for the early diagnosis of bone sarcoma ; that the operation wound in osteomyelitis should be closed. The fact that the book has already reached a third edition is evidence that it is appreciated by many students and practitioners.

**Phrenicectomy.** By Richard Cory, M.B., Ch.B. Jamaica Central Board of Health. (No date, no price.)—Many Bristol men will remember the author during his student days at the University and the Bristol Royal Infirmary. He is now in practice at Kingston, Jamaica. This little book is a most practical account of how to avulse the phrenic nerve for pulmonary tuberculosis, and giving full details of the anatomy and the difficulties that may arise during operation. The author describes the indications and contra-indications for the operation. The work is clearly illustrated by home-made sketches, and bespeaks wide experience.

**A Guide to the Surgical Paper, with Questions and Answers.** By R. J. McNeill Love, M.S., F.R.C.S. Pp. 78. London : H. K. Lewis & Co. Ltd. 1935. Price 5s.—The purpose of this small volume of seventy-seven pages is explained by the title, and a further indication of this is contained in a short preface. A brief introduction is positively crammed with useful information and suggestions in regard to the best methods of imparting to the examiner in surgery what knowledge the student possesses, with a minimum of effort and annoyance to both the parties concerned. Then follow twenty-eight questions, well chosen to cover a wide field of surgical knowledge, and the remaining pages are devoted to the answering of these questions in model but skeleton form ; the leaves are sealed. The book should appeal to the "poor examination student," to whom it could be of the greatest assistance, and although brevity must have been the author's intention, the reader feels that just one complete answer would add considerably to its value.

**The Osteopathic Lesion.** By George Macdonald, M.B., Ch.B., and W. Hargrave-Wilson. Pp. xii. 141. Illustrated. London : William Heinemann Ltd. 1935. Price 7s. 6d.—This small book represents an attempt on the part of men conversant with anatomy and physiology to justify the main concept of osteopaths *i.e.* "the spinal lesion."

It presents a refreshing contrast to the general run of osteopathic literature in the absence of the wild generalizations and impossible claims that the latter has put forward. Gross dislocation of joints are denied. The suggestion of pressure on vessels and nerves in the inter-vertebral foramina is scouted, and an attempt is made by an appeal to nerve reflexes, the obscure functions of the sympathetic nervous system and bio-chemistry to explain the phenomena of osteopathy. The mysterious spinal lesion is defined as a joint strain either acute or chronic, and it is frankly admitted that nothing objective can be demonstrated post-mortem or by operative exposure. It is admitted that irregularity of the spinous processes is no guide, and that in most cases the lesion cannot be demonstrated by X-rays. We are left, therefore, with the single physical sign of tenderness in or around a certain spinal segment as the sole objective evidence of "the lesion." Certain arresting statements are made which it would be interesting to put to the proof. It is claimed, for example, that if six patients suffering from dyspepsia were ranged with six normal individuals, the former could be recognized by osteopathic examination of the spine by finding the lesion in the joints between the fifth and sixth dorsal vertebrae. The book is an earnest attempt to give a scientific basis for the osteopathic cult, and as such is of some interest to those who believe that in the midst of a mass of rubbish there does exist a germ of truth in the cult of osteopathy.

**The Student's Pocket Prescriber.** By D. M. MACDONALD, M.D., D.P.H. Tenth Edition. Pp. 263. Edinburgh: E. & S. Livingstone. 1934. Price 3s.—The fact that this little book has reached a tenth edition is clear proof that students have found it a useful aid in the art of prescribing. In addition to various prescriptions, there is a useful appendix with such inclusions as a diet list, weights and tables, incubation table of zymotic diseases, posological tables and a vocabulary for prescriptions.

**Incompatibility in Prescriptions and How to Avoid It.** Fourth Edition. By THOMAS STEPHENSON, D.Sc., Ph.C., F.R.S.E., F.C.S. Pp. viii. 62. Edinburgh: The Prescriber Publishing Offices. 1935. Price 6s.—This book is truly a *vade mecum* for the newly-qualified man, and may well find a place on the bookshelves of the new resident house physician and house surgeon, the general practitioner, and the pharmacist.

The outstanding qualifications of the author to appreciate the difficulties of prescriber and dispenser are unquestionable. The book is divided into two parts, the first dealing with the principles of chemical, physical and therapeutic incompatibility and the second dealing with the individual incompatibilities of drugs in the form of an alphabetical list. The author has dealt with his subject with masterly thoroughness, and one can hardly think of an incompatible prescription not anticipated in the book. One notices with pleasure under "precipitation of alkaloids" a clear and concise note on the limits of strychnine possible to be dispensed with alkalies. Also the note on the advisability of prescribing hexamine and acid phosphate of sodium in separate mixtures. In Part II the behaviour of the individual drugs is very completely treated. One misses any reference to the miscibility of paraldehyde with fatty oils, the miscibility with volatile oils being of relatively little importance. Monographs dealing with glycine, hexyl-resorcinol and bismuth-sodium-tartrate are evidence of the recognition of the more recently adopted *materia medica*. Certainly a book to be bought by every newly-qualified man.

**Common Skin Diseases.** By A. C. ROXBURGH, M.D., F.R.C.P. Third Edition. Pp. xxxii., 377. Illustrated. London: H. K. Lewis & Co. Ltd. 1936. Price 15s.—The appearance of a third edition of this text-book is sufficient proof of its popularity. Very few additions and alterations have been made, but notes on gold dermatitis, Besnier's prurigo, recurrent cellulitis and diet in lupus vulgaris have been added; there are also several new illustrations. Another new feature of this edition is that prescriptions are given in percentages and metric quantities as well as in apothecaries' weights and measures. This book is quite one of the best, if not the best, of the smaller text-books on dermatology, and has won for itself the established place which we predicted for it in our review of the first edition.

